# Impact of the COVID-19 outbreak and interventions on hand, foot and mouth disease in Zhengzhou, China, 2014–2022: a retrospective study

**DOI:** 10.1186/s12879-024-09244-w

**Published:** 2024-04-09

**Authors:** Wanyu Jia, Xue Zhang, Ruiyang Sun, Peng Li, Chunlan Song

**Affiliations:** Children’s Hospital Affiliated to Zhengzhou University, Henan Children’s Hospital, Zhengzhou Children’s Hospital, No. 1, South University Road, Erqi District, 450018 Zhengzhou, China

**Keywords:** COVID-19, Hand, foot and mouth disease, Autoregressive integrated moving average model

## Abstract

**Background:**

Since December 2019, COVID-19 has spread rapidly around the world, and studies have shown that measures to prevent COVID-19 can largely reduce the spread of other infectious diseases. This study explored the impact of the COVID-19 outbreak and interventions on the incidence of HFMD.

**Methods:**

We gathered data on the prevalence of HFMD from the Children’s Hospital Affiliated to Zhengzhou University. An autoregressive integrated moving average model was constructed using HFMD incidence data from 2014 to 2019, the number of cases predicted from 2020 to 2022 was predicted, and the predicted values were compared with the actual measurements.

**Results:**

From January 2014 to October 2022, the Children’s Hospital of Zhengzhou University admitted 103,995 children with HFMD. The average number of cases of HFMD from 2020 to 2022 was 4,946, a significant decrease from 14,859 cases from 2014 to 2019. We confirmed the best ARIMA (2,0,0) (1,1,0)_12_ model. From 2020 to 2022, the yearly number of cases decreased by 46.58%, 75.54%, and 66.16%, respectively, compared with the forecasted incidence. Trends in incidence across sexes and ages displayed patterns similar to those overall.

**Conclusions:**

The COVID-19 outbreak and interventions reduced the incidence of HFMD compared to that before the outbreak. Strengthening public health interventions remains a priority in the prevention of HFMD.

## Introduction

In Wuhan, China, on December 31, 2019, a new variety of atypical pneumonia named “COVID-19” brought on by SARS-CoV-2 was first discovered [1, 2]. Later, COVID-19 quickly led to a worldwide pandemic outbreak, which caused significant health and socioeconomic damage due to its clinical severity and ease of transmission [3].

Responding to an outbreak of COVID-19, China put a ban on travel to and from Wuhan city and launched a national emergency response on January 23, 2020 [4]. Due to the rapid response and strict epidemic control measures, the COVID-19 epidemic in China was brought under control in a short period of time. Subsequently, due to the lack of readily available and effective pharmaceutical agents and the highly infectious nature of the new coronavirus, epidemic prevention and control entered a normalized phase. To contain or slow the spread of SARS-CoV-2, countries have implemented various nonpharmaceutical interventions (NPIs). These measures include maintaining restrictions on social activities, such as issuing stay-at-home orders, canceling large gatherings, restricting domestic and international travel, and strengthening education on personal protective measures, for example, promoting the wearing of masks, maintaining good hand hygiene and breathing etiquette [5, 6].

With the exception of COVID-19, China has a large population, and the incidence of other major infectious diseases is among the highest in the world [7]. A growing number of studies suggest that nonpharmacological interventions might play a beneficial role in delaying the temporal impact of a pandemic, reducing overall and peak incidence. Several studies have shown that measures to prevent COVID-19 can largely reduce the spread of other infectious diseases, especially respiratory-transmissible diseases, such as influenza and tuberculosis [6–9]. Hand, foot and mouth disease (HFMD) is a common pediatric contagious disease caused by Enterovirus (EV) infection and is more prevalent in children under 5 years. Close contact is a principal mode of transmission of HFMD, and it can also be transmitted through respiratory droplets; drinking or eating water and food contaminated with the virus can also cause infection [10, 11]. HFMD is a global disease, and China has a high incidence of HFMD throughout the year [12, 13]. The incidence of HFMD in China was similarly affected during the COVID-19 outbreak.

Henan Province is located in central China, with 17 prefecture-level cities and 1 province-administered county-level city, with a resident population of nearly 100 million, the third largest population in China. Henan is one of the most serious HFMD epidemic provinces in China [14], and Zhengzhou is the capital city of Henan Province. Children’s Hospital Affiliated to Zhengzhou University is the largest tertiary level A pediatric hospital in Henan and a sentinel hospital for surveillance of hand, foot and mouth disease and promotion and implementation centers of prevention and treatment for HFMD designated by the Chinese state. Analyzing the impact of nonpharmaceutical COVID-19 interventions on the incidence of other infectious diseases is of great significance for public health and infectious disease supervision in China. This study collected and discussed the incidence data of HFMD in Children’s Hospital Affiliated to Zhengzhou University before and after the COVID-19 epidemic, in order to serve as a reference point for future work on controlling, preventing, and monitoring infectious diseases.

## Methods

### Data Collection

Using electronic medical record data from the Children’s Hospital Affiliated to Zhengzhou University which is the largest specialized hospital for children in Henan Province, an uncontrolled before-and-after study was carried out. The information of all HFMD patients in the disease surveillance report management system of the Children’s Hospital Affiliated to Zhengzhou University was collected, and the data meeting the relevant requirements were exported. There was no intervention on human participants in our study.

Diagnostic criteria for children with HFMD: ① Age less than 18 years, ② Clinical manifestations of HFMD, such as fever, rash on the hands, feet, mouth, buttocks and so on; ③ Positive nucleic acid test specific for enterovirus (CV-A16, EV-A71 and so on).

All patients who were diagnosed with HFMD between January 2014 and December 2022 were enrolled in this study. The entire study phase was then divided into three subphases to assess the reduction in HFMD admission rates: the “Pre-epidemic period” from 2014 to 2019, the “Post-epidemic lockdown period” from January to June 2020, and the “Post-epidemic normalization period” from April to December 2022.

### Statistical analysis

#### ARIMA model

The autoregressive integrated moving average (ARIMA) model is a valuable method for assessing the influence of large-scale interventions. ARIMA is a time-series forecasting tool and is a common statistical method for predicting the short-term impact and trends of acute infectious diseases. ARIMA can take into account seasonality, autocorrelation, and underlying trends and enables flexible modeling of various impacts [15]. The ARIMA (p, d, q) model extends the autoregressive (AR), moving average (MA), and ARMA models [16]. With this approach, we are able to decompose the randomness, stationarity, and seasonality of the time series and select a suitable forecasting model based on the time series analysis. Due to the seasonality of the HFMD, we utilized a seasonal ARIMA model, and the model constructed herein was written as (p, d, q) (P, D, Q) s [15, 17]. The parameters p, d, and q indicate the order of the AR, which indicates the degree of variation of the original time series, and the order of the MA and D, D, Q, and s indicate the seasonal autoregression, seasonal integration, seasonal moving average, and seasonal period length, respectively [6]. The ARIMA model was built using the monthly number of admissions.

We built seasonal autoregressive integrated moving average (SARIMA) models to fit HFMD activity from 2014 to 2019 and predict HFMD epidemic levels during 2019–2022 under a counterfactual scenario in which the COVID-19 pandemic did not occur; therefore, strict NPIs were not used. SPSS 26.0 statistical software was used for the statistical analysis of the experimental data. *P*<0.05 indicated that the difference was statistically significant.

## Results

### Characteristics of the study subjects

**Fig. 1:**
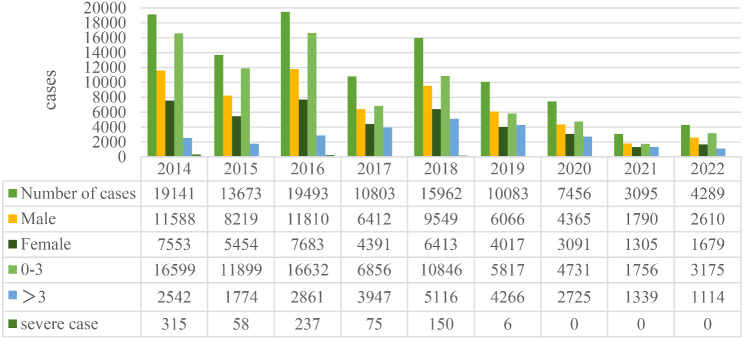
Cases of HFMD from November 2014 to December 2022 in Henan, China

**Fig. 2:**
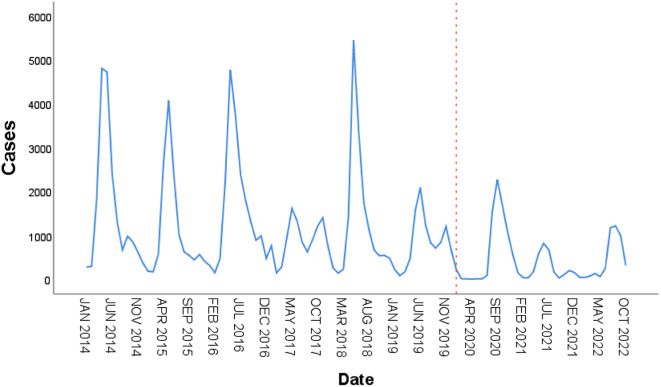
Monthly cases of HFMD from January 2014 to December 2022 in Henan

From January 1, 2014, to October 31, 2022, Children’s Hospital of Zhengzhou University admitted 103,995 children with hand, foot and mouth disease, including 62,409 males and 41,586 females. The male-to-female ratio was approximately 1.5:1, which is basically consistent with previous studies. The age of HFMD admissions in our hospital was concentrated in people aged three years and below, with a total of 78,311, accounting for 75.3% of the total number of cases. The specific number of admissions per year is shown in Fig. 1. As shown in Fig. 2, from 2014 to 2019, the peak incidence of HFMD was concentrated from May to July, with obvious seasonality. In contrast, there was a significant delay in the peak incidence of HFMD from 2020 to 2022.

### ARIMA models specification

**Fig. 3:**
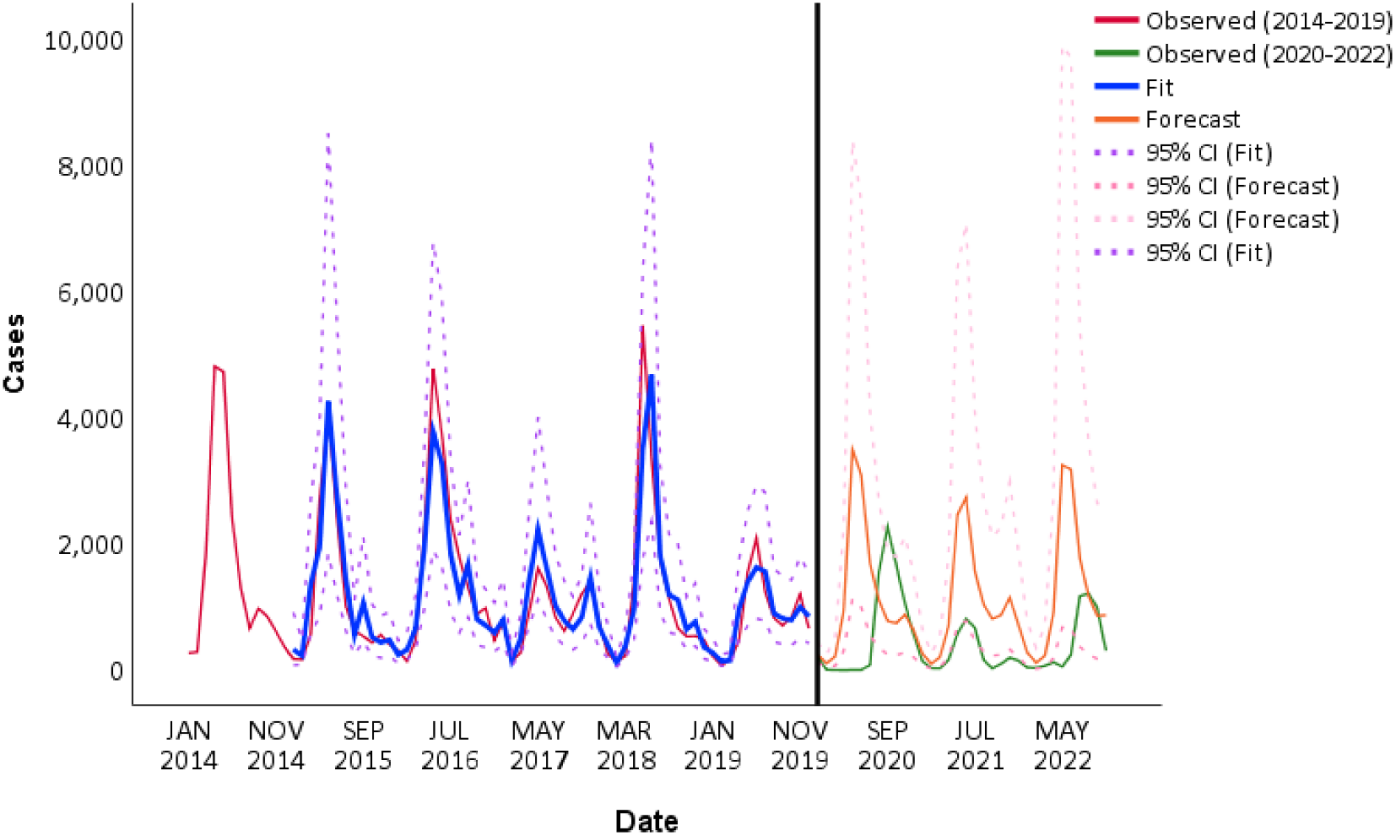
Observations and predictions of the ARIMA (2,0,0) (1,1,0)_12_ model

We used data from January 2014 to December 2019 to build an ARIMA model. First, since the original time series was nonstationary, to induce stationarity, the original time series should be transformed by a logarithmic algorithm, difference (d) and seasonal difference (D). We specified values for d = 0 and D = 1 (due to the presence of seasonality). Second, the random, stationary and seasonal effects on the time-series data were analyzed using the autocorrelation function (ACF) and partial autocorrelation function (PACF) analysis methods. Third, the most appropriate model can be selected using parameter estimation and model testing. To compare the models’ goodness-of-fit, diagnostic parameters, including the coefficient of determination (R^2^), normalized Bayesian Information Criterion (BIC) and Ljung-Box test, were used. And *P*<0.05 means the difference is statistically significant. As a consequence, using the goodness-of-fit test statistics, we confirmed the optimal ARIMA (2,0,0) × (1,1,0)_12_ model, the highest R^2^ was 0.85, the BIC value was 12.526, and the residual error sequence was a white-noise sequence. Finally, the model was used to forecast the incidence of HFMD between January 2020 and October 2022 under a counterfactual scenario. (Fig. 3)

### Changes in HFMD incidence

We compared the mean number of HFMD cases from 2020 to 2022 (4946) with that from 2014 to 2019 (14,859) and found that the number of HFMD cases decreased significantly from 2020 to 2022. At the same time, the peak incidence of HFMD in 2020 and 2022 is significantly delayed, from the original May to July to August to October. Meanwhile, the incidence of severe HFMD had a downward trend, with only 6 cases of severe HFMD in 2019, while no further cases of severe HFMD have occurred since the outbreak.

We found that the observed HFMD activity levels from 2020 to 2022 were significantly lower than predicted. As shown in Fig. 3, during the post-epidemic lockdown period, the incidence of HFMD significantly decreased and even decreased to single digits. Compared with the forecast rates under the counterfactual scenario, the incidence in the post-epidemic lockdown period declined by 96.8%. The number of cases in February 2020 declined by 103 cases and declined by 209 cases in March 2020. The number of cases dropped by 103 in January, 209 in February, 209 in March, 954 in April, 3,482 in May and 3,072 in June 2020. During the post-epidemic normalization period, the number of observed cases of HFMD increased sharply from July to November 2020, and by August 2020, the number of patients exceeded the number predicted by the ARIMA model but was still lower than that predicted in terms of full-year data. The number of total 7456 patients with HFMD in 2020 decreased by 46.58% compared with the number forecasted by the ARIMA model. During the post-epidemic normalization period, the numbers of patients with HFMD in 2021 and 2022 were 3095 and 4289, respectively, while the numbers forecasted by the ARIMA model were 12,652 and 12,676, representing decreases of 75.54% and 66.16%, respectively.

### Stratified analysis by sex and age

**Fig. 4:**
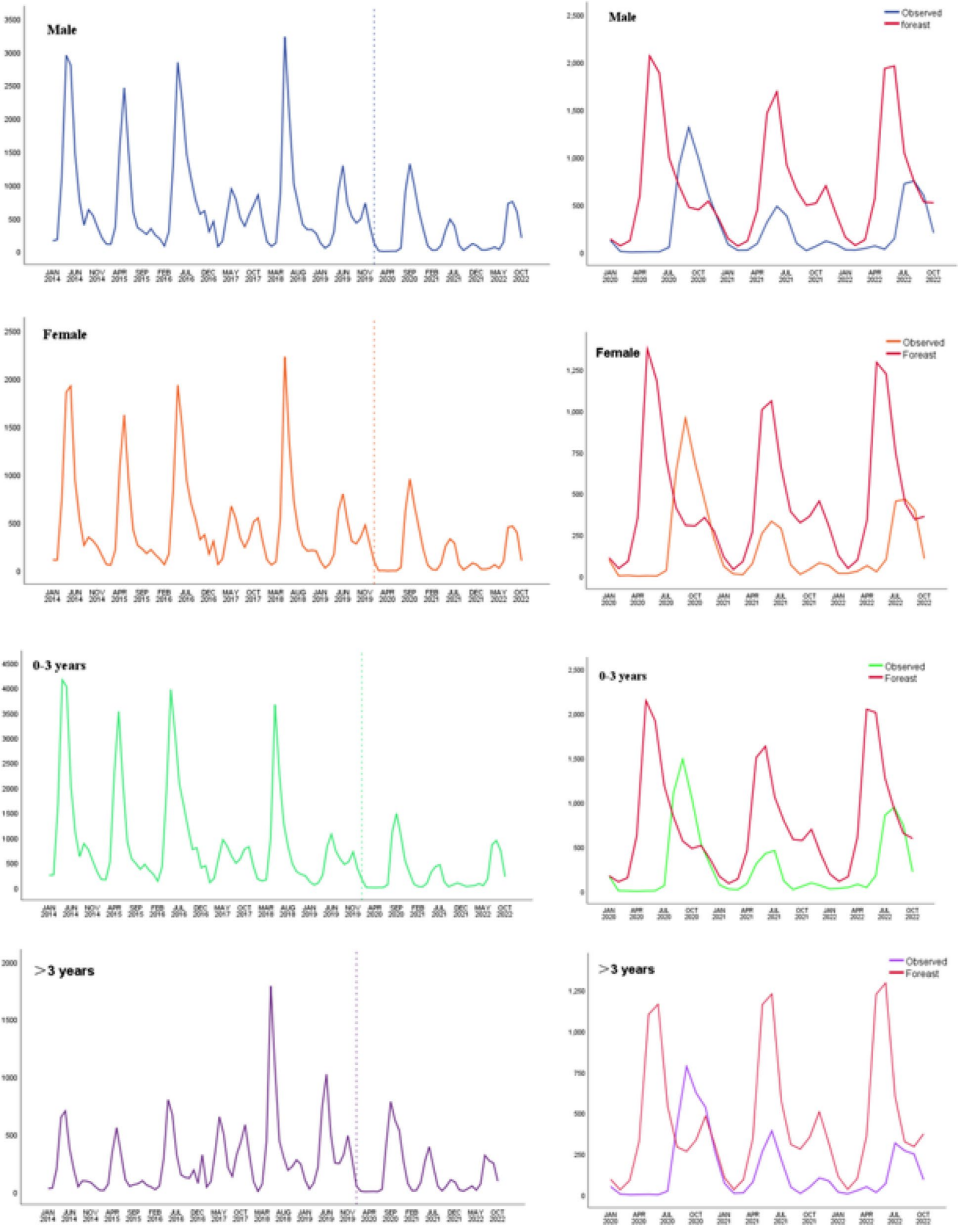
Stratified analysis by sex, age

Figure 4 represents the monthly cases of HFMD with different sex and age groups from 2014 to 2022, as well as the observations and predictions of HFMD in 2020–2022 stratified by sex and age. The incidence of HFMD across sexes and ages followed the same patterns as the overall trends.

## Discussion

In this study, we found that the mean incidence of HFMD decreased by 66.71% from 2020 to 2022 compared to the average from 2014 to 2019 in Henan, China. In addition, the incidence of HFMD has been almost zero since January 2020 and has remained at near zero for 6 consecutive months. Compared to the predicted values, the annual incidence rates from January 2020 to October 2022 decreased by 46.58-75.54%, respectively.

Studies have revealed a decreasing trend in the incidence of most statutory infectious diseases in China, with NPIs playing a positive role in the prevention and control of respiratory and intestinal infectious diseases [7, 18]. A number of studies have shown that during the COVID-19 pandemic, NPIs and behavioral modifications to prevent COVID-19 could have impacted the dynamics of influenza, tuberculosis, and other infectious disease transmission throughout the world [6, 19–21]. The incidence and spread of HFMD were greatly reduced as a result of NPIs and other preventative and control measures for COVID-19, according to our findings, which were largely in line with those of previous research. While most of the previous studies explored only the short-term effects of the epidemic on infectious diseases [22], this study explored the changes in the incidence of HFMD in the two years following the epidemic and clarified the long-term effects of the epidemic on the incidence of HFMD.

The incidence of HFMD practically vanished during the post-epidemic lockdown period (from January to June 2020), when the impact of COVID-19 on the disease was at its maximum. This happened as a result of the government enforcing a blockade and limiting inhabitants’ access to the outside world. HFMD is mainly transmitted through close contact, and nurseries and schools are the main HFMD transmission sites [23]. In this period, the government took mandatory measures to restrict tourism activities, close schools, work from home, and reduce the use of public transportation, which significantly reduced people’s movement and inhibited the spread of HFMD from the transmission route. There was a brief rebound in the incidence of HFMD after the government’s mandatory lockdown policy was lifted, and the prevention and control of the COVID-19 epidemic entered a normalized phase, which should be attributed to the opening of childcare centers and schools, as well as increased movement of people. In terms of long-term impacts, however, the incidence of HFMD remains lower than that in the pre-epidemic period. Additionally, since we are a provincial children’s hospital and some of our children originate from outside of our city, travel restrictions and embargoes related to COVID-19 may have been one of the reasons for the decline in the number of cases during COVID-19.

In post-epidemic normalization period, the government took routine preventive and control measures, including promoting personal hygiene practices, maintaining social distancing, practicing hand hygiene, wearing masks, enforcing place codes and health codes, and performing nucleic acid testing. During this period, the HFMD incidence decreased significantly from the predicted value but still showed significant seasonality. The peak incidence of HFMD in 2021 was the same as that before the COVID-19 outbreak, while the peak incidence in 2022 was delayed, which may be related to the different levels of closure and control measures taken by the Henan Provincial Government according to the outbreak. The potential long-term impact of COVID-19 is dependent on changes in public health awareness and hygiene practices. During the post-epidemic normalization period, it had become an essential part of daily life for people to consciously focus on personal hygiene, wearing masks and maintaining hand hygiene.

In the Asia-Pacific region, including China, HFMD is a serious disease burden in children. And HFMD outbreaks are common in kindergartens and primary schools [24, 25]. Children can readily contract HFMD pathogens both within and outside of the classroom by touching infected hands, personal items, toys, tableware, and bedding. The EV-A71 vaccine, which was launched in China in 2016, is currently the only effective means of preventing hand, foot and mouth disease [26]. However, due to the low vacctionation rate of the vaccine and its non-protective effect against HFMD caused by CV-A6 or other viruses [27], NPIs remain the most important measures for preventing the spread of HFMD. Handwashing and personal hygiene by children and their caregivers were found to have a significant protective effect against HFMD in studies [28, 29]. During the COVID-19 epidemic, closing kindergartens and schools, extending holidays and improving hand hygiene education for children and their caregivers were effective methods for not only containing the spread of COVID-19 but also controlling the epidemic of HFMD.

On December 26, 2022, the National Health Commission of the People’s Republic of China issued an announcement renaming novel coronavirus pneumonia to novel coronavirus infection. With the approval of the State Council of China, effective January 8, 2023, preventive and control measures for novel coronavirus infection as a Class A infectious disease under the law of the People’s Republic of China on prevention and control of infectious diseases will be lifted; under the Law of the People’s Republic of China on Health and Quarantine at the State Border, novel coronavirus infection will no longer be included in the management of quarantine infectious diseases. China’s epidemic prevention policy has undergone large changes, with no more routine nucleic acid testing, no more restrictions on interregional travel, and no more school lockdowns due to the epidemic. In this case, the incidence of various infectious diseases, such as hand, foot, and mouth disease, may increase compared to the previous level, which is a challenge for disease prevention and control departments and pediatric medical workers. However, since personal hygiene measures such as wearing masks to maintain hand hygiene are well established, the incidence of HFMD may not rise again to the pre-epidemic level. The findings of this study also confirm that the NPI is highly important for preventing the spread of HFMD and that the government and hospitals should still insist on education and emphasize the importance of hand hygiene and other measures for the population.

Our study has a few limitations. First, we examined only medical files from a single institution. However, the Children’s Hospital Affiliated to Zhengzhou University is the largest pediatric specialist hospital in Zhengzhou, and it is the designated specialist for the treatment of HFMD in Zhengzhou, so the number of cases is representative. Second, while ARIMA is a mature and applicable technology for forecasting infectious disease, selecting the most appropriate ARIMA model is subjective and challenging. Moreover, previous studies have indicated that HFMD incidence is significantly influenced by climatic factors [30]. However, in this study, we did not include the effects of meteorological factors or other potential factors on the incidence of HFMD, which may have influenced the results.

## Conclusion

According to this study, the incidence of HFMD has decreased compared to the previous one as a result of the COVID-19 epidemic and interventions. NPIs such as mask use, avoiding close contact, and hand cleaning were effective in halting the spread of HFMD. For the prevention and management of HFMD, strengthening public health measures continues to be a top goal.

## Data Availability

No datasets were generated or analysed during the current study.
